# Supplementary material for: Myositis-Specific and Myositis-Associated Antibodies in Fibromyalgia Patients: A Prospective Study
Source: Biomedicines. 2023 Feb 22;11(3):658. doi: 10.3390/biomedicines11030658 (PMC10045737; doi:10.3390/biomedicines11030658)
Supplement: Supplementary file 1 [file biomedicines-11-00658-s001.zip › biomedicines-2211593-supplementary.pdf]

**code**	code
age	age
**gender**	gender
WPI	Widespread Pain Index
SSS	Symptoms Severity Score
TP	Tender Points
AF	Atrial Fibrillation
**other arry**	other arrythmyas
**dyslipidem**	dyslipidemia
**hypetensi**	hypetension
**GERD**	Gastro-esophageal reflux disease
**hypothyr**	hypothyroidism
**depressio**	depression
**T2DM**	Type2 Diabetes Mellitus
**MI**	History of Myocardial Infarction
**stroke**	History of stroke
**previous**	previous cancer
**kind**	kind of cancer
**anemia**	anemia
**25OHD**	vitamin D
**ALP**	Alkaline Phosphatase
**creatinine**	creatinine
**proteinur**	proteinuria spot
**complem**	low fractions of complement
**CRP**	C Reactive Protein
**ESR**	Erythrosedimentation Rate
**TSH**	Tyroid Stimulating Hormone
**fT3**	Triiodothyronine free
**fT4**	Tetraodothyronine free
**AST**	Aspartate transaminase
**ALT**	Alanine Transaminase
**LDH**	Lactic Dehydrogenase
**CPK**	Creatine Phosphokinase
**MSA**	Myositis Specific Antibodies
**MAA**	Myositis Assocaited Antibodies
**ANA dic**	ANA dichotomous
**ANA**	Antinuclear Antibodies
**speckled**	speckled
**cito**	citoplasmic pattern
**homo**	homogeneous pattern
**nucl**	nucleolar pattern
**combine**	combined
**Jo1**	anti-Jo1
**RF**	Rheumatoid factor >2x ULN
**SSA60K**	anti-SSa60Kd
**KU**	anti-Ku
**Pm/scl**	anti-Pm/scl
**Tif1gamm**	anti-Tif1gamma
**Mi2**	anti-Mi2
**PL7**	anti-PL7
**MDA5**	anti-MDA5

**SAE**	anti-SAE
**PL12**	anti-PL12
**OJ**	anti-OJ
**histones**	anti-histones
**ACPA**	anti citrullinated protein antibody
**DsDNA**	anti-DsDNA
**RNP**	anti-RNP
**Ro52**	anti-Ro52kD
**SRP**	anti-SRP
**EMG**	Electhro-myography (positive for myopathy)
**HRTC**	High Resolution Computed Tomography
**ST**	Schirmer Test
**UWST**	Untimulated Whole Saliva Test
**CTD**	Connective Tissue Disease
**months C**	months to from the baseline to the development of CTD
**Other Dia**	Other Diagnoses
**months t**	months to the recognition of other diagnoses
**Spine MR**	Spine Magnetic Resonance Imaging
**evolution**	evolution
